# Regional differences in unmet need for contraception in Kenya: insights from survey data

**DOI:** 10.1186/s12905-015-0240-z

**Published:** 2015-10-14

**Authors:** Sam W. Wafula

**Affiliations:** Sexual and Reproductive Health Program, Population Council, Nairobi, Kenya

**Keywords:** Unmet need, Contraception, Pregnancy, Kenya, Reproductive health

## Abstract

**Background:**

Women are described as experiencing unmet need for contraception if they are fecund, sexually active and wish to postpone or limit childbearing but fail to use contraception to do so. The consequences of unmet need include unwanted pregnancy, induced abortions, school dropout due to pregnancy and premature maternal deaths. Global efforts aimed at addressing the adverse effects of unmet need abound. In Kenya, one in every four married women in the reproductive age bracket (15–49 years) has unmet need for contraception. Regional differences exist but the reasons behind these differences remain poorly understood. The purpose of this study was to examine the extent to which regional differentials in unmet need for contraception exists and to explain the regional differences in unmet need for contraception in Kenya.

**Methods:**

The paper used the Kenya Demographic and Health Survey (2008/09) data. Unmet need for contraception was measured based on the revised estimates contained in the survey. Summary statistics were used to show the percentage differences in the values of selected covariates across the high and low unmet need zones. The dependent variable had three categories: no unmet need, unmet need for spacing and unmet need for limiting births. The categorical nature of this dependent variable which is not ordered in any way lends itself to the use of multinomial logistic regression. The paper applied the seemingly unrelated estimation *(suest)* test to ascertain whether the covariate coefficients between the high and low unmet need zones were different. Stata Version 13.0 was used for analysis.

**Results:**

The percentage values of the selected covariates of unmet need for contraception were much higher in the high unmet need zone as compared to those observed in the low unmet need zones. On the overall, 15.4 % of women in the high unmet need zone had unmet need to space their next birth as compared to 8.6 % of their counterparts. Likewise, the percentage of women who wanted to limit further births stood at 14.1 % among women residing in high unmet need zones while those in low unmet need zones had 10.5 %. Further analysis based on seemingly unrelated estimation found that in general, a comparison of the coefficients been the high and low unmet need regions were significantly different (*p* < 0.05).

**Conclusion:**

Evidence from the nationally representative KDHS 2008/09 shows that regional differentials in the covariates of unmet need for contraception exist. There is need to address religious inhibitions that stymie contraceptive uptake especially in the high unmet need regions. Efforts should promote maternal education and economically empower women in order to reinforce individual and contextual attitudes towards the benefits of contraception. The government should also establish social franchise programs to increase access to costly long acting and permanent methods of contraception to poor women.

## Background

Worldwide, over 222 million women have unmet need for contraception [1]. Women are described as experiencing unmet need for contraception if they are fecund, sexually active and wish to postpone or limit further childbearing but fail to use contraception for one reason or another [2, 3]. Unmet need for contraception denies women the economic and social benefits of family planning and violates their reproductive health right on the number and timing of childbirths.

**Fig. 1:**
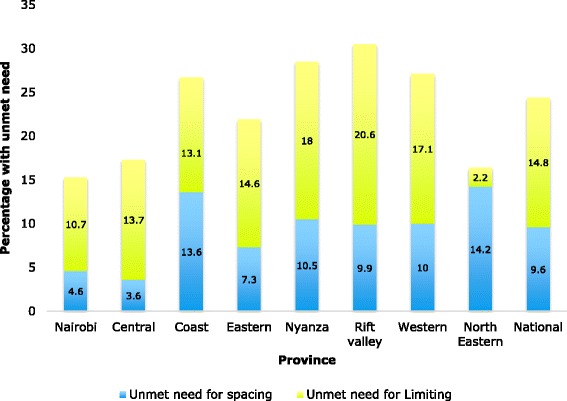
Percentage distribution of unmet need to space and limit births by province and national level, KDHS 2008/09

In Kenya, a quarter of the married women in the reproductive age group (15–49 years) have unmet need for contraception [4]. Addressing unmet need for family planning improves the livelihood of a household by reducing poverty and empowering women [3]. At the macro level, tackling unmet need can accelerate fertility and mortality decline by limiting unintended births which may otherwise end in abortions and maternal deaths [3, 5]. Further, investments aimed at promoting voluntary family planning initiatives can mob up the huge gap in unmet need by increasing access [3].

## Methods

This study used data drawn from the 2008/09 KDHS. The data are national in scope. Out of the 8444 women of reproductive age (15–49 years) that were interviewed during the 2008/09 KDHS, 5041 were either currently married women or were in a union. This paper analyses KDHS data for women who were married or in union due to the following reasons: first, this cohort is the most sexually active with the highest risk of experiencing unmet need and its adverse consequences. Secondly, married women or those in union are bound to face more opposition from their spouses in their decision to use family planning as compared to the rest. Finally, the methodology for estimating unmet need for contraception among married women or those in union is the most developed of them all and is widely used globally [1, 6].

Unmet need for family planning is computed from women’s fertility preference and current contraceptive behaviour. Married women who were pregnant were asked whether they wanted to get pregnant then. Those who wanted to become pregnant were categorized as not having unmet need for contraception. Among those who did not want to get pregnant then, they were asked whether they wanted to get pregnant later (after 2 years or more) or not at all. Those who wanted to get pregnant later were categorized as having unmet need to space while those who did not want the pregnancy at all were categorised as having unmet need to limit births.

On the other hand, married women who were sexually active and were fecund but were not using any contraceptives were asked whether they wanted to get pregnant during their most recent pregnancy (within 5 years). Those who wished they could have postponed their last pregnancy by at least 2 years were categorized as having unmet need to space while those who did not want to get pregnant at all were classified as having unmet need to limit. Married women who were currently pregnant or not were also asked about their timing and future intentions of becoming pregnant. Out of all these questions, a measure was computed to categorize women who had met needs for contraception as well as unmet need for spacing and limiting further births. A detailed algorithm for the computation of unmet need estimates is available in earlier works on this subject [1].

In this study, a regional approach was adopted. Specifically, regions that had a higher than national rate of unmet need for contraception were categorized as high unmet need zones while their counterparts were categorized as low unmet need zones. Using this categorization, Rift Valley, Nyanza, Western and Coast provinces which had unmet need levels of 30.3 % were grouped as high unmet need zones since their rate exceeded the national rate of 25.6 %. On the other hand, Nairobi, Central, Eastern and North Eastern provinces were grouped as low unmet need zones since they only had unmet need levels of 18 % against the national average of 25.6 %. Details of unmet need for contraception for each province are shown in Fig. 1. I employed the difference in difference estimates to illustrate the differences in the levels of unmet need between the high and low unmet need regions. This is shown in Table 1.

**Table 1:** Regional percentage change in unmet need in Kenya, KDHS 2008/09

	UNS_H_(a)	UNS_L_(b)	UNL_H_(c)	UNL_L_(d)	Difference in difference (DiD) estimates *DiD = (a + c)-(b + d)*
Total	15.4	8.6	14.1	10.5	10.4
Maternal age					
15–24	26.1	20	6.1	5	7.2
25–29	19.8	11.4	11.7	7.3	12.8
30–39	11.8	5.6	15.6	12.3	9.5
40–49	1.9	1.4	25.5	15.4	10.6
Number of living children					
0–2	19.1	10.5	5.6	3.8	10.4
3 to 5	15.3	8	15.4	13.6	9.1
6+	8.2	4.4	29	23.6	9.2
Maternal education					
None	18.2	14.5	10.3	5.7	8.3
Primary incomplete	18.4	10.2	17.7	16.6	9.3
Primary complete	15.5	9.8	15.4	11.6	9.5
Secondary +	14.3	11	22.4	16.6	9.1
Spousal education					
None	17.3	14	12.7	4	12
Primary	17.3	10.4	16	12.5	10.4
Secondary+	21.4	12.4	21.5	18.5	12
Household wealth index					
Poor	12	14	17.7	16	−0.3
Middle	19.4	6.1	12.3	10.8	14.8
Rich	13.6	7.2	11.2	8	9.6
Religion					
Roman catholic	18.8	7.2	15.8	13.8	13.6
Protestant/other Christian	14	8	14.5	10.2	10.3
Muslims	18.6	16.4	7.5	2.8	6.9
Reads newspapers					
Not at all	16.5	9.7	14.8	11.5	10.1
Less than once a week	17.5	8.3	11.9	11.3	9.8
At least once a week	11.4	6.3	15.2	7.2	13.1
Almost every day	3.7	4.2	9.3	6.7	2.1
Listens to radio					
Not at all	19.1	15	14.4	9.1	9.4
Less than once a week	16.5	10	14	16.1	4.4
At least once a week	19.7	9.8	11.8	11.9	9.8
Almost every day	13.5	6.7	14.6	9.7	11.7
Watches television					
Not at all	17.6	11.6	15.7	12.4	9.3
Less than once a week	16.1	8.4	10.9	12.3	6.3
At least once a week	14.7	8.2	6.5	6.9	6.1
Almost every day	8.2	4.5	13.1	7.9	8.9

*UNS*_*H*_ unmet need to space in the high zones, *UNS*_*L*_ unmet need to space in the low zones, *UNL*_*H*_ unmet need to limit in the high zones, *UNL*_*L*_ unmet need to limit in the low zones

A multinomial logistic regression was applied in each of the two unmet need zones to assess the net effect of the covariates on unmet need status. This is an appropriate statistical procedure since the dependent variable has more than two unordered outcomes namely: women without unmet need, women with unmet need to space and women with unmet need to limit births. Using women without unmet need as the base outcome category, this study assessed the significance of the selected covariates on unmet need to space and to limit further births. Separate regression models were fitted for both the low and high unmet need zones. A one unit increase in any of the independent variables either increased or decreased the relative log odds of experiencing unmet need to space or limit vis-à-vis our base outcome (no unmet need).

The fitted multinomial regression models were then compared using the seemingly unrelated estimation (*suest*) command in Stata version 13.0. The purpose of this comparison was to assess whether differences in the covariate coefficients existed between the high and low unmet need zones. Explanatory variables used in this study were categorized based on theory as well as the conventional practice. For instance, young women are more likely to use contraceptives for spacing while older women prefer methods that limit childbearing since they have already achieved their desired family size. The categories thus reflect these theoretical underpinnings. The categories used are as follows:-

- Respondent’s age (15–24; 25–29; 30–39 and 40–49 years).
- Number of living children (0–2; 3–5; 6 or more children)
- Maternal education (None, Primary incomplete, Primary complete; Secondary and above)
- Spousal education (None, Primary; Secondary and above)
- Respondent’s religion (Roman Catholic; Protestant/other Christian, Islam)
- Household wealth index (0: poor, 1: middle and 2: the rich).

The household wealth status was computed using the principal component analysis (PCA) and the factor weights of the first component were used to place households in either poor, middle or rich category. Past studies show that the above factors are associated with unmet need for contraception [6, 7]. 

The ethical approval for KDHS 2008/09 was obtained from the Kenya Medical and Research Institute (KEMRI). Written informed consent was sought from eligible clients before administration of the survey. The author also formerly obtained permission to use KDHS data from MEASURE DHS which are freely available once permission is granted. The data are available on the following website: http://www.measuredhs.com.

## Results

Table 1 shows the results of bivariate analysis across the zones. Zones with high unmet need for contraception had generally higher percentage points of unmet need for spacing and limiting as compared to the rates observed in low unmet need zones. In both the high and low unmet need zones, unmet need for spacing births decreased with increasing age while unmet need to limit further births increased with age. This pattern was maintained when the existing number of living children was assessed against unmet need for spacing and limiting across the two unmet need zones. As parity increases, unmet need to space births tends to plummet while the need to limit further births tends to increase. In particular, women with at least 6 children tended to express the greatest unmet need to limit further childbearing than the rest.

The more educated the woman, the lower the likelihood she would experience unmet need to space or limit further childbearing across the two zones. This pattern was however not true when the education of spouse was considered. Table 1 shows that spouses with the highest level of education (tertiary) had the highest unmet need to space or limit births in both the high and low unmet need zones as compared to the rest.

Women who are poor are generally expected to exhibit the highest level of unmet need for contraception to space or limit births. While results from this study generally support this assertion, an exception was noted among poor women in the high zones where they enjoyed lower rates of unmet need for spacing as compared to those in the middle class and the rich.

Women who were Protestants enjoyed the lowest unmet need for spacing irrespective of the zone. Those professing the Roman Catholic faith had the highest unmet need for spacing in the high zone while Muslims had the highest unmet need for spacing in the low unmet need zone. In addition, Catholics generally had the highest unmet need for limiting births irrespective of the zone while Muslim women had the least. Women who read newspapers almost daily and those who listened to radio or watched television daily exhibited the lowest unmet need for spacing and limiting across the two zones.

### Determinants of unmet need status in the high unmet need zone: a multinomial logistic regression analysis

Table 2 shows that the effect of education on unmet need was in the expected direction.

**Table 2:** Multinomial log odds of selected covariates on the unmet need in the high unmet need region, KDHS 2008/09

Unmet need to space versus no unmet need				Unmet need to limit births versus no unmet need		
Maternal age	(β)	Exp (β)	CI	(β)	Exp (β)	CI
45–49 (Ref)						
15–19	3.687	39.92	(3.14, 4.23)	−0.025	0.976	(−.80, 0.75)
20–24	3.428	30.81	(2.98, 3.86)	−0.114	0.892	(−.41, 0.19)
25–29	2.981	19.71	(2.55, 3.40)	−0.209	0.811	(−.43, .010)
30–34	2.715	15.11	(2.29, 3.13)	−0.079	0.924	(−.27, 0.11)
35–39	1.965	7.13	(1.53, 2.39)	0.399	1.491	(0.23, 0.57)
40–44	0.360	1.43	(−.18, .90)	0.723	2.061	(0.56, 0.89)
Number of living children						
0–2 (Ref)						
3 to 5	0.166	1.180	(−.01, .34)	1.059	2.883	(.80, 1.32)
6+	0.259	1.296	(.030, .49)	1.632	5.113	(1.35, 1.91)
Place of residence						
Rural (Ref)						
Urban	−0.082	0.922	(−.29, .13)	−0.105	0.901	(−.30, 0.89)
Maternal education						
Secondary complete + (Ref)						
None	0.472	1.603	(.15, −.80)	0.193	1.212	(−0.09, −0.47)
Primary incomplete	0.449	1.567	(.17, −.73)	0.720	2.055	(0.46, 0.97)
Primary complete	0.454	1.575	(.18, −.73)	0.730	2.074	(.49, .97)
Secondary incomplete	0.133	1.142	(−.21, −.47)	0.580	1.786	(.30, .86)
Spousal education						
Secondary complete + (Ref)						
None	0.185	1.202	(−.06, .43)	−0.198	0.821	(−.42, 0.02)
Primary	0.069	1.072	(.09, .22)	0.272	1.313	(0.13, 0.41)
Secondary incomplete	−0.488	0.614	(−.86 –.11)	0.287	1.333	(0.03, 0.55)
Household wealth index						
Poor (Ref)						
Middle	−0.331	0.718	(−.49, −.16)	−0.594	0.552	(−.74, −.45)
Rich	−0.576	0.562	(−.69, −.38)	−0.301	0.740	(−.46, −.14)
Religion						
Protestants/other christians (Ref)						
Catholics	0.350	1.419	(−.63, −.24)	−0.439	0.645	(−.63, −.24)
Muslims	0.455	1.576	(−.37, .21)	−0.080	0.923	(−.39, .21)

A one unit increase in education among women with primary education in the high unmet need zone increased the multinomial log odds of experiencing unmet need for spacing by 0.45 relative to women with no unmet need. Married women in the 15–19 year age bracket were 3.68 times more likely to experience unmet need for spacing relative to those without unmet need holding other factors constant. The effect of partners’ education on unmet need for spacing was interesting. Women whose partners had no formal education were 0.08 points less likely to experience unmet need for spacing as compared to those without unmet need holding other factors constant. This association was however not statistically significant (*p* > 0.05). On the contrary, women whose partners had incomplete secondary level of education were 0.48 points less likely to experience unmet need for spacing as compared to our base outcome and this association was statistically significant (*p*-value < 0.05).

As expected, women in the middle or rich class exhibited lower log odds of experiencing unmet need to space births compared to those without unmet need. Muslims and Catholics were significantly associated with increased log odds of experiencing unmet need for spacing births.

On the contrary, women who had primary level of education or above were more likely to experience increased log odds of experiencing unmet need to limit further births and this association was statistically significant (*p*-value < 0.05). Apart from women aged 35–39 and 40–44 years old, the rest were not significantly associated with increased log odds of experiencing unmet need to limit further births relative to those without unmet need.

The higher the number of living children, the more likely a married woman was bound to have increased log odds of having unmet need to limit further births. Partners’ educational level was also associated with increased log odds of experiencing unmet need to limit births. For instance, women whose spouses had attained primary level of education were 1.3 times (i.e. exp (0.27)) more likely to have unmet need to limit childbearing relative to our base outcome category.

One of the interesting results from this study is that in the high unmet need zones, women who were in the middle class or the rich were less likely to experience the log odds to limit further childbearing. Further, women who profess the Islamic faith were significantly less likely to experience the log odds of unmet need to limit births.

### A multinomial logistic regression analysis of the determinants of unmet need status in the Low unmet need zone

Table 3 shows the results of the covariates of unmet need to space and limit in the low unmet need zone. The dummies of the respondent’s educational attainment were associated with increased log odds of unmet need to space births as compared to the base category. For instance, women with no formal schooling were 3.8 times more likely to experience unmet need to space births relative to women who did not have unmet need. Married women in all the reproductive age groups were significantly associated with increased log odds of experiencing unmet need to space births relative to those without unmet need.

**Table 3:** Multinomial log odds of selected covariates on unmet need for contraception for low unmet need regions, KDHS 2008/09

	Unmet need to space births versus no unmet need for contraception			Unmet need to limit births versus no unmet need for contraception		
Maternal age	(β)	Exp(β)	CI	(β)	Exp(β)	CI
45–49 (Ref)						
15–19	2.48	11.89	(1.56, 3.4)	−13.88	0.00	(−17.5 17.7)
20–24	3.07	21.56	(2.54, 3.6)	0.246	1.28	(−.25 .74)
25–29	2.26	9.62	(1.78, 2.76)	−0.15	0.86	(−.51 .21)
30–34	2.10	8.17	(1.63, 2.57)	0.027	1.03	(−.25 .30)
35–39	1.17	3.23	(0.69, 1.65)	0.495	1.64	(.25 .74)
40–44	0.90	2.46	(0.38, 1.42)	0.117	1.12	(−.14 .38)
Number of living children						
0–2 (Ref)						
3–5	0.16	1.17	(−.11, 0.43)	0.966	2.63	(.63 1.30)
6+	0.69	1.98	(0.34, 1.04)	1.931	6.90	1.55 2.31
Place of residence						
Urban (Ref)						
Rural	−0.63	0.53	(−0.93, −0.33)	−0.584	0.56	(−0.85, −0.32)
Maternal education						
Secondary complete + (Ref)						
None	1.34	3.82	(0.79, 1.89)	−0.333	0.72	(−0.80, 0.14)
Primary incomplete	0.83	2.29	(0.35, 1.31)	0.383	1.47	
Primary complete	0.89	2.44	(0.43, 1.35)	0.262	1.30	(−.05, 0.57)
Secondary incomplete	0.23	1.26	(−0.47, 0.93)	0.116	1.12	(−0.26, 0.5)
Paternal education						
Secondary complete + (Ref)						
None	0.29	1.34	(−0.13, 0.71)	−1.124	0.32	(−1.53, −0.72)
Primary	0.64	1.89	(0.29, 0.98)	−0.301	0.74	(−.51, −.10)
Secondary incomplete+	1.40	4.06	(0.97, 1.84)	−0.384	0.68	(−.75, −.02)
Household wealth index						
Poor (Ref)						
Middle	−0.23	0.79	(−0.53, .07)	−0.762	0.47	(−.99, −.53)
Rich	−0.61	0.54	(−0.94, −0.29)	−0.809	0.45	(−1.06, −.56)
Religion						
Protestant/other christian (ref)						
Roman catholic	0.36	1.44	(0.09, 0.64)	0.301	1.35	(0.11, 0.49)
Muslims	0.23	1.25	(−0.13, 0.59)	−1.153	0.32	(−1.56, −0.74)

Women with at least 6 children were 1.3 times more likely to experience unmet need to space their next birth as compared to women with no unmet need. Place of residence was not significantly associated with the log odds of experiencing unmet need to space. Women whose spouses had secondary education had 0.6 times less chances of experiencing unmet need for spacing relative to the base category. Women in the middle class and the rich were less likely to experience the log odds of unmet need to space relative to the base category. Religion was significantly associated with increased log odds of experiencing unmet need to space in the low unmet need zones.

The effect of maternal education appears to wane in the low unmet need zones in relation to limiting further births. A part from women with primary education, the rest were not significantly associated with unmet need to limit births. Equally, if we discount women who were aged 40–45 years, the rest were not significantly associated with unmet need for limiting births relative to our base outcome category. Parity remained an important predictor for experiencing unmet need to limit births. For instance, women who had at least 6 children or more were 6.9 times more likely to experience unmet need to limit further childbearing. Women residing in urban areas had reduced log odds of experiencing unmet need to limit births. Spousal education was a critical predictor of unmet need to limit births. Education of the spouse generally had a negative effect on the woman’s log odds of experiencing unmet need to limit births relative to women without unmet need. Household wealth index as well as religious affiliation were also important predictors of unmet need for birth limiting.

### Are the covariates for unmet need to space and limit births different between the high and low unmet need zones?

In the assessment of whether the covariates were significantly different between the high and low unmet need zones, the seemingly unrelated estimation (*suest*) test was applied using Stata version 13.0. This test presents simultaneous results across the two zones (low and high unmet need zones) and determines whether the coefficients of the two zones are the same or not. If the coefficients of the factors do not differ between the two zones, then we would expect that the *p*-value would be >0.05 and the inverse is also true.

In order to carry out the *suest* command, multinomial regression models were run separately for both the high and low unmet need zones and the models were stored for comparison using a post estimation command in Stata 13.0 called the *est store*. This was followed by a comparison of the models using *suest* command. Finally, the overall models were compared using the following test command in Stata: *test [Model1 = Model2].* The preceding test command compared whether the covariate coefficients for unmet need for spacing and limiting were similar or not between the high and low unmet need zones if women with no need for contraception were used as the base outcome.

The overall results indicated that in general, the coefficients of the selected covariates for unmet need for spacing and limit further childbearing were indeed significantly different between the high and low unmet need zones (χ^2^ test = 1065.13; df = 21; *p*-value < 0.001 and χ^2^ test = 1062.13; df = 20; *p*-value < 0.001 respectively). Table 4 presents the results for this test. A detailed exposition on the application of this method in found in Stata guidelines [7].

**Table 4:** A comparison of the coefficients between the high and low unmet need regions: The application of seemingly unrelated estimation regression

	Model 2 versus model 1		Model 4 versus model 3	
	(Unmet need to space in high vs low unmet need zones)		(Unmet need to limit in high vs low unmet need zones)	
Maternal age	Model 2 (β)	Model 1 (β)	Model 4 (β)	Model 3 (β)
45–49 (Ref)				
15–19	3.49^a^	-.018	2.50^a^	−13.86^a^
20–24	3.43^a^	-.112	3.11^a^	.269
25–29	2.98^a^	-.203	2.28^a^	-.139
30–34	2.72^a^	-.077	2.12^a^	.034
35–39	1.97^a^	.403^a^	1.19^a^	.490^a^
40–44	0.36	.727^a^	.914^a^	.124
Number of living children				
0–2 (Ref)				
3 to 5	0.17	1.05^a^	.119	.892^a^
6+	0.26^a^	1.62^a^	.651^a^	1.86^a^
Place of residence				
Rural (Ref)				
Urban	0.47^b^	.187	1.31^a^	-.387
Maternal education				
Secondary complete + (Ref)				
None	0.45^a^	.714^a^	.785^a^	.353^b^
Primary incomplete	0.45^a^	.726^a^	.830^a^	.189
Primary complete	0.13	.576^a^	.177	.113
Secondary incomplete	0.18	-.194	.227	−1.17^a^
Spousal education				
Secondary complete + (Ref)				
None	0.07	.273^a^	.625^a^	-.314^b^
Primary	−0.49^b^	.287^b^	1.47^a^	-.292
Secondary incomplete	−0.33^a^	-.590^a^	-.204	-.750^a^
Household wealth index				
Poor (Ref)				
Middle	−0.58^a^	-.257^a^	-.264	-.562^a^
Rich	0.33^a^	.062	-.204	.272^b^
Religion				
Protestants/other (Ref)				
Roman catholic	0.35^a^	-.424^a^	-.264	−1.06^a^
Muslims	0.45^a^	-.076	.332^b^	1.31^a^
*χ* ^*2*^ *test*		*1065.13*		*1062.6*
*df*		*21*		*20*
*p-value*		*0.000*		*0.000*

^a^ *p* < 0.000

^b^ *p* < 0.05

The key results from this comparison reveals that by and large, the covariate coefficients of the regression models between the two zones were significantly different (*p*-value < 0.05). For instance, apart from women with secondary education, the coefficients of unmet need to space for the rest of the dummies of education were significantly different between the two regions. This was also largely true for maternal age with the exception of those aged 40–44 years and above. Women with 0–2 children were not in any way different between the two zones in so far as unmet need to space or limit births was concerned. The coefficients for the place of residence as well as spousal educational level were also not significant in explaining the regional differences in unmet need for spacing. Unmet need to limit births did not differ between regions when spouses who did not have any formal education was analysed. Household wealth index and religion were by and large significantly different in explaining unmet need to space between the two regions (*p* < 0.05).

Apart from women with no formal education, the coefficients of other educational categories that were associated with explaining unmet need to limit were significantly different. Before age 40–44 years, the coefficients of maternal age were not significantly different between the two regions in so far as unmet need to limit childbearing was concerned.

## Discussion

The purpose of this study was to assess whether regional differentials in unmet need for contraception exist. The survey data drawn from KDHS 2008/09 were used. Results show striking evidence of regional variations in the level of unmet need for contraception. The covariates behind the difference in the regional unmet need estimates were statistically significant. In both zones, religion played an important role in accounting for the observed differences in unmet need for contraception to space and limit births. Maternal age, maternal education, household wealth index and number of living children a woman has were the other factors that significantly accounted for the regional differences in unmet need for contraception.

Unmet need for spacing births decreased with increasing age while unmet need to limit further births increased with age. Past studies have also found similar results. Normally, young mothers are hardly through with child bearing and hence would prefer short term methods of contraception to space their next births. Consequently, these mothers tend to have high levels of unmet need for spacing. However, as they age, most tend to achieve their ideal family sizes and tend to prefer long term and in some instances, permanent methods of contraception. Equally, as the number of children increase, women tend to experience unmet need to limit further births. This is because they tend to attain their ideal family sizes and hence are more likely to use contraceptives to limit having additional children [7, 8].

Religion was significantly important in explaining regional levels of unmet need to space and limit. The influence of religion takes several mechanisms. First, religion can generate self-perceptions and healthy beliefs which may impact either positively or negatively on the uptake of contraceptives [7]. For instance, there is a very high opposition among Catholics and Muslims in the uptake of contraceptives and hence women belonging to these religions tend to exhibit high unmet need for spacing and limiting further births as compared to the rest. Point estimates from this study showed that the percentage of Muslims and Catholics is higher in the high unmet need region as compared to the low unmet need region and this difference is statistically significant (Odds ratio 1.2; *p* < 0.05). This could account for the high unmet need levels in the high as opposed to low unmet need regions.

The high unmet need among women with low level of education could be due to their deep rooted misconceptions about the benefits of contraception. Such women tend to have strong negative attitudes towards contraception including perceived side effects [7].

## Conclusion

In reducing regional differentials in unmet need for contraception, it is critical to promote the uptake of contraceptives. However, some methods of contraception including long acting and permanent methods of contraception are not free of charge in Kenya and thus poor women may experience unmet need for contraception due to their inability to access such methods. There is need for development partners in collaboration with the government to establish social franchise programs with a view to increase access to contraception methods among poor women who would otherwise not afford such methods. This would enable poor women to postpone their next birth or limit further childbearing.

Efforts to address religious inhibitions that stymie contraceptive use could focus on community sensitization programs on the benefits of family planning. Additionally, there is need to promote maternal education as well as economically empowering women to enable them access contraception. Such efforts will alter individual and contextual attitudes towards the benefits of family planning.

Finally, intervention packages that aim to boost family planning use should take cognizance of the existing regional differences in unmet need for contraception by adopting optimal region specific strategies. The strategies should address regional specific barriers such as religious and cultural beliefs that could undermine contraceptive uptake.
